# Identification and Characterization of a Novel Thermostable GDSL-Type Lipase from *Geobacillus thermocatenulatus*

**DOI:** 10.4014/jmb.2012.12036

**Published:** 2021-02-12

**Authors:** Eunhye Jo, Jihye Kim, Areum Lee, Keumok Moon, Jaeho Cha

**Affiliations:** 1 Department of Microbiology, College of Natural Sciences, Pusan National University, Busan 46241, Republic of Korea; 2 Microbiological Resource Research Institute, Pusan National University, Busan 46241, Republic of Korea

**Keywords:** Extremophiles, *Geobacillus*, GDSL esterase/lipase, thermophiles, enzyme characterization

## Abstract

Two putative genes, *lip29* and *est29*, encoding lipolytic enzymes from the thermophilic bacterium *Geobacillus thermocatenulatus* KCTC 3921 were cloned and overexpressed in *Escherichia coli*. The recombinant Lip29 and Est29 were purified 67.3-fold to homogeneity with specific activity of 2.27 U/mg and recovery of 5.8% and 14.4-fold with specific activity of 0.92 U/mg and recovery of 1.3%, respectively. The molecular mass of each purified enzyme was estimated to be 29 kDa by SDSPAGE. The alignment analysis of amino acid sequences revealed that both enzymes belonged to GDSL lipase/esterase family including conserved blocks with SGNH catalytic residues which was mainly identified in plants before. While Est29 showed high specificity toward short-chain fatty acids (C4-C8), Lip29 showed strong lipolytic activity to long-chain fatty acids (C12-C16). The optimal activity of Lip29 toward *p*-nitrophenyl palmitate as a substrate was observed at 50°C and pH 9.5, respectively, and its activity was maintained more than 24 h at optimal temperatures, indicating that Lip29 was thermostable. Lip29 exhibited high tolerance against detergents and metal ions. The homology modeling and substrate docking revealed that the long-chain substrates showed the greatest binding affinity toward enzyme. Based on the biochemical and in silico analyses, we present for the first time a GDSL-type lipase in the thermophilic bacteria group.

## Introduction

Lipases are glycerol ester hydrolases (E.C. 3.1.1.3) that catalyze the hydrolysis of triacylglycerols to free fatty acids and glycerol. They resemble esterases (E.C. 3.1.1.1) in catalytic activity but differ in that substrates. True lipases prefer water-insoluble fats containing medium- to long-chain fatty acids. Lipases are used extensively in the detergent, food, dairy, pulp, and pharmaceutical industries due to their high productivity and diversity, such as substrate specificity, stability in organic solvents, and high degree of regioselectivity [1].

Bacterial lipases are classified into eight families based on amino acid sequence homology [2]. Family I lipases, called true lipases, are large group which is further divided into 6 subfamilies. They possess the pentapeptide Gly-Xaa-Ser-Xaa-Gly (GxSxG) motif with the active site serine situated near the center of the conserved sequence [2, 3]. Most of the bacterial lipases from *Bacillus* and *Staphyloccocus* species belongs to this family. The enzymes grouped in family II do not exhibit the conventional GxSxG motif but rather display a Gly-Asp-Ser-Leu (GDSL) motif containing the active site serine residue. The GDSL motif localized in near N-terminus of amino acid sequence which is compared to GxSxG motif conserved in center of the sequence [4]. GDSL lipases represent the lipolytic activities with multifunctional properties and broad substrate specificity [5, 6]. Furthermore, a subgroup of this GDSL family was classified as the SGNH hydrolase superfamily, with four conserved residues Ser, Gly, Asn and His in four conserved blocks I, II, III, and V [6]. While the SGNH hydrolases are well known in eukaryotic organisms, the isolation and characterization of SGNH hydrolases from bacteria remain to be limited [7]. In bacteria, GDSL motif enzymes are generally known as esterase type which has preference to short chain fatty acids [8-11]. To date, there have been few reports of GDSL family lipases in bacteria [12, 13]. One example is a GDSL lipase from *Mycobacterium tuberculosis* and it was known to be actively involved in the intracellular survival during the nutritive stress conditions [12].

*Geobacillus* species, which belongs to thermophilic Gram-positive spore-forming bacteria that can grow over a range of 45-75°C, are of interest for biotechnology field as source of thermostable enzymes. Also, *Geobacillus* species are known to have potential availability for digesters of lignocellulose, hydrocarbons bioremediators, biofuel producers, cellular factories for heterologous expression of enzymes because of their structural and functional stability in extreme environments [14-17]. Several lipases which belong to family I have been reported from this species [18, 19]. A number of family I and II esterases from this species have been characterized [20, 21] including the GDSL esterase which showed the highest activity to *p*-nitrophenyl butyrate at optimal conditions [9]. To our knowledge, however, there have been no reports of thermostable GDSL-type lipases that favor long fatty acid chains.

In this study, two lipolytic enzymes, Est29 and Lip29, from *Geobacillus thermocatenulatus* KCTC3921 was cloned, purified, and characterized. With comprehensive in silico and wet lab experiments our work present that Lip29 is a first member of GDSL family lipase found in the thermophilic bacteria.

## Materials and Methods

### Chemicals and Reagents

The *p*-nitrophenyl acetate (*p*NP-C2), *p*-nitrophenyl butyrate (*p*NP-C4), *p*-nitrophenyl hexanoate (*p*NP-C6), *p*-nitrophenyl octanoate (*p*NP-C8), *p*-nitrophenyl dodecanoate (*p*NP-C12), *p*-nitrophenyl myristate (*p*NP-C14), *p*-nitrophenyl palmitate (*p*NP-C16) and *p*-nitrophenyl stearate (*p*NP-C18) were purchased from Sigma-Aldrich (USA). The Bio-Rad Protein Assay Dye Reagent used in the Bradford assay was from Bio-Rad (USA). The nTaq and PrimeStar HS DNA polymerases for polymerase chain reaction (PCR) were purchased from Enzynomics (Korea) and TaKaRa Bio (Japan), respectively. T4 DNA ligase and restriction enzymes were purchased from TaKaRa Bio. SDS, Tween 20, and Tween 80 were purchased from Affymetrix/USB (USA). DMSO was purchased from Junsei (Tokyo). Triton X-100 was purchased from Pharmacia (Sweden). All other chemicals in this study were purchased from Sigma-Aldrich.

### Construction of the Est29 and Lip29 Expression Vector

The genomic DNA of *Geobacillus thermocatenulatus* KCTC3921 was kindly provided from the Food Biotechnology Laboratory at Yonsei University, Korea. Based on the sequence in the KEGG database (http://www.genome.jp/kegg/), the gene of Est29 (798 bp) was amplified by PCR with two primers: Est29_F (5'-GTCATATGGGCCGGAGAGTTG-3') and Est29_R (5'-GTTCTCGAGTTGTTCACCCTC-3') containing NdeI and XhoI restriction sites, respectively. Lip29 gene (783 bp) was amplified by PCR with other two primers: Lip29_F (5'- AACATATGAAACGATGGGGATGG-3') and Lip29_R (5'- CCCTCGAGTTGCAGCAAACTCCC-3') containing same restriction sites of Est29. The restriction enzyme sites were underlined. The amplification was performed by PCR with Prime STAR polymerase. The conditions of PCR were as follows: one cycle of denaturation at 98°C for 5 min, 30 cycles of denaturation at 98°C for 10 sec, annealing at 55°C for 5 sec, extension at 72°C for 50 sec, and final extension step at 72°C for 5 min. The two PCR products were ligated with T-Blunt vector (Biofact, Daejeon, Korea) and transformed into *E. coli* DH5α competent cells using heat shock transformation protocol. The transformants were plated onto LB plate containing ampicillin (100 μg/ml) and incubated at 37°C overnight. After preparation of constructed plasmid, the recombinant plasmids were cleaved with respective NdeI and XhoI restriction enzymes, and the digested fragments were ligated into the pET21a vector (Novagen Inc., Madison, WI, USA) previously digested with the same restriction enzymes. These recombinant expression plasmids were transformed into *Escherichia coli* BL21(DE3) for overexpression of the esterase and lipase genes.

### Sequence Alignment with Other GDSL Family Lipase/Esterase

To compare the conserved motifs on amino acid sequence, the peptide sequences of the characterized bacterial esterases/lipases containing GDSL-motif were searched on NCBI (https://www.ncbi.nlm.nih.gov/) and PDB protein databases (https://www.rcsb.org/). The secondary structure prediction and alignment were conducted using ClustalW (https://embnet.vital-it.ch/software/ClustalW.html) and ESPript 3.0 web server (http://espript.ibcp.fr/ESPript/cgi-bin/ESPript.cgi). After multiple sequence alignment using phylogeny software (http://www.phylogeny.fr/index.cgi), the amino acid sequences are reshaped by GeneDoc (http://genedoc.software.informer.com/2.7/). The members include TesA of *Pseudomonas aeruginosa* (PDB: 4JGG), arylesterase of *Thauera* sp. (Accession No. WP_038010598), GDSL-family esterase of *Geobacillus thermodenitrificans* T2 (Accession No. ACD02023), and SGNH hydrolase family esterase of *Bacillus* sp. K91 (Accession No. ALI97418).

### Purification of the Recombinant Est29 and Lip29

The recombinant *E. coli* BL21(DE3) strains were grown in LB broth with ampicillin (100 μg/ml) overnight at 37°C. The cultured cells (5 ml) were transferred to 500 ml LB medium with ampicillin and cultured at 37°C until cultures reached an optical density at 600 nm of 0.4-0.5. The cells were induced with 0.1 mM isopropyl β-D-1-thiogalactopyranoside (IPTG), and cultures were incubated with shaking at 30°C for 4 h. After 4 h induction, the cells were harvested by centrifugation (4,500 ×*g*, 50 min, 4°C) and resuspended in buffer (20 mM sodium phosphate pH 7.4, 0.5 M NaCl, and 20 mM imidazole). Cells were lysed by sonication and the crude cell extracts were centrifuged (2,000 ×*g*, 10 min, 4°C) to remove cell debris. The supernatants were then centrifuged (20,000 ×*g*, 40 min, 4°C) for the removal of insoluble proteins. The soluble proteins were then incubated at 60°C for 15 min to remove the thermolabile proteins of *E. coli* from the cell extracts. The resulting supernatants were filtered and loaded to nickel-nitrilotriacetic acid (Ni-NTA) affinity chromatography column. The bound proteins were washed with 50 mM imidazole in the same buffer (20 mM sodium phosphate pH 7.4, 0.5 M NaCl, and 50 mM imidazole) and eluted using 200 mM imidazole buffer (20 mM sodium phosphate pH 7.4, 0.5 M NaCl, and 200 mM imidazole). Fractions of 1 ml were collected and protein concentrations were determined by the Bradford method [22]. The molecular mass of the purified enzyme was determined by SDS-PAGE with DokDo-MARK broad range marker (ELPIS biotech, Korea). After SDS-PAGE for analysis of the fractions containing recombinant protein, dialysis was performed using a dialysis membrane (MWCO 6-8000) and the purified enzymes were stored at 4°C.

### Enzyme Assay

Lipase activity on *p*NP-C16 and esterase activity on *p*NP-C8 as a substrate was determined by measuring the amount of *p*NP released after the addition of the enzyme [23, 24]. The standard assay was conducted at 60°C for 15 min in a final volume of 100 μl of a reaction mixture containing 50 mM sodium phosphate buffer (pH 7.0), 1mM *p*NP-C16 or *p*NP-C8 dissolved in isopropyl alcohol, and 1 μg of purified enzyme. The reaction was stopped by the adding 100 μl of 1 M Na_2_CO_3_. The liberated *p*NP was quantified at 420 nm using a microplate spectrophotometer (Epoch, Biotek, USA). One unit (1 U) of lipase activity was defined as the amount of enzyme needed to liberate 1 μmol of *p*NP per minute at the conditions described above.

### Substrate Specificity

The substrate specificity of the enzyme was determined on *p*NP esters with different length of acyl chain (C2-C18). The reaction was carried out under the standard conditions and the concentration of each substrate was 10 mM.

### Effect of pH and Temperature on the Enzyme Activity

The enzyme activity was determined at 60°C for 15 min with *p*NP-C16 (Lip29) or *p*NP-C8 (Est29) as substrate over a pH range of 3.0-9.0 in the following buffers (50 mM final concentration): sodium acetate (pH 3.0-6.0); sodium phosphate (pH 6.0-8.0); Tris-HCl (pH 8.0-9.0); bicarbonate-carbonate (pH 9.0-10.5). The temperature dependence of the enzyme activity was determined over a range of 30 to 100°C in 50 mM bicarbonate-carbonate buffer, pH 9.5 (Lip29) or 50 mM sodium phosphate buffer, pH 6.0 (Est29). The thermostability was evaluated by measuring the remaining activity after incubation of the purified enzyme at 50-90°C for various intervals of time up to 3 h. Aliquots were withdrawn at the indicated times and assayed on *p*NP substrate at standard conditions.

### Effect of Metal Ions and Detergents on the Lipase Activity

The effect of metal ions on the enzyme activity was evaluated using optimal assay conditions as described above. The purified enzyme was pre-incubated with each of the selected metal ions at final concentration of 1 mM at 50°C for 5 min prior to lipase assay. The lipolytic activity of the purified enzyme without metal ions was considered relatively as 100%. The activity of the purified enzymes towards various detergents was also measured by using dimethyl sulfoxide (DMSO), sodium dodecyl sulfate (SDS), Triton X-100, Tween 80 and Tween 20 at final concentrations of 1%. The enzyme was pre-incubated with each of the detergent at the standard conditions for 5 min prior to activity measurement. The residual activity was measured according to the standard assay conditions, and the activity without addition of the detergent was defined as 100%.

### Homology Modeling and Molecular Docking Analysis

The amino acid sequences of Est29 and Lip29 were obtained using Expasy translation tool (https://web.expasy.org/translate/). The 3D structure modeling of the two enzymes was conducted by Phyre2 [25] and screened using PyMol software (https://pymol.org/2/) to compare structure using the lysophospholipase TesA from *Pseudomonas aeruginosa* (PDB entry 4JGG) as a template. AutoDock Vina in PyRx virtual screening tool PyRx 0.8 was used for docking the substrate compounds with the enzymes and calculating the binding affinity [26].

## Results

### Identification and Analysis of Genes Encoding a GDSL Family Lipase/Esterase in *G. thermocatenulatus* KCTC3921

**Fig. 1:**
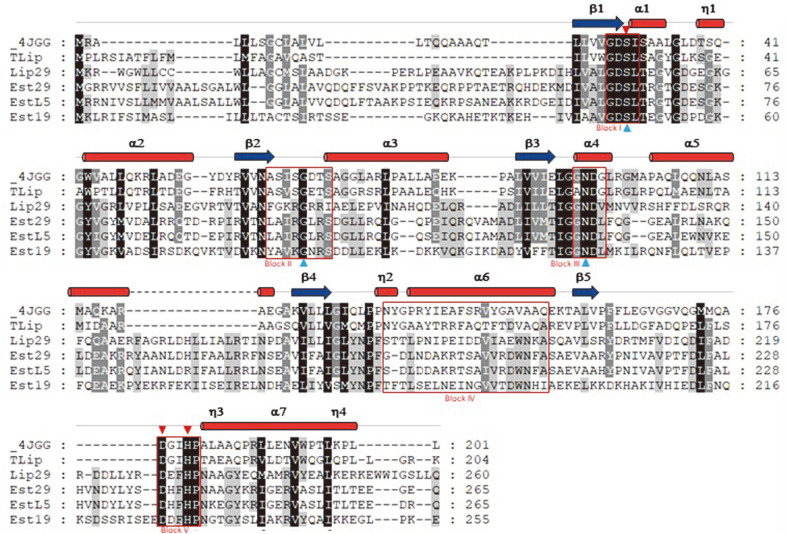
Multiple sequence alignment of Lip29 and Est29 with other GDSL family of esterases and lipases. Secondary structures including α-helices and β-sheets are shown based on 4JGG. Four conserved blocks of I, II, III, IV, and V are boxed. The black and gray blocks show the identical and similar sequences, respectively. Closed triangles and closed inverted triangles indicate amino acid residues conserved in the catalytic triad and oxyanion hole, respectively. 4JGG: GDSLfamily lysophospholipase of *Pseudomonas aeruginosa*, Tlip: arylesterase of *Thauera* sp. (Accession No. WP_038010598), EstL5: GDSL-family esterase of *Geobacillus thermodenitrificans* T2 (Accession No. ACD02023), Est19: SGNH hydrolase family esterase of *Bacillus* sp. K91 (Accession No. ALI97418).

Two putative lipase/acylhydrolases with GDSL-like motif (GT3921_11225 and GT3921_10635) were found in the genome sequence of *G. thermocatenulatus* KCTC3921. The open reading frame (ORF) of these two genes, designated to Est29 and Lip29, is composed of 798 and 783 nucleotides corresponding to 265 and 260 amino acids, respectively. The conserved domain search of Est29 and Lip29 from NCBI revealed that both putative enzymes contained the SGNH-hydrolase YpmR-like domain which belonged to SGNH-hydrolase superfamily of lipases and esterases. BlastP search analysis of both enzymes revealed identities from 74.7% to 95.5% with various hypothetical GDSL family lipases/esterases belonging to *Geobacillus* spp. and *Bacillus* spp. Multiple sequence alignment of Est29 and Lip29 with other GDSL lipase/esterases revealed that Est29 and Lip29 possessed conserved GDSL motif near N-terminus (Fig. 1). Five consensus sequence (I–V) and four invariant important catalytic residues Ser, Gly, Asn, and His in blocks I, II, III, and V, respectively, indicate that they are also classified SGNH-hydrolase superfamily. The serine, aspartate and histidine triads consist oxyanion hole supported glycine and asparagine residues as proton donor [6]. Although thermostable GDSL family esterases from *Geobacillus* have been characterized [9], GDSL-type lipases have not been characterized, yet.

### Expression and Purification of Est29 and Lip29

**Fig. 2:**
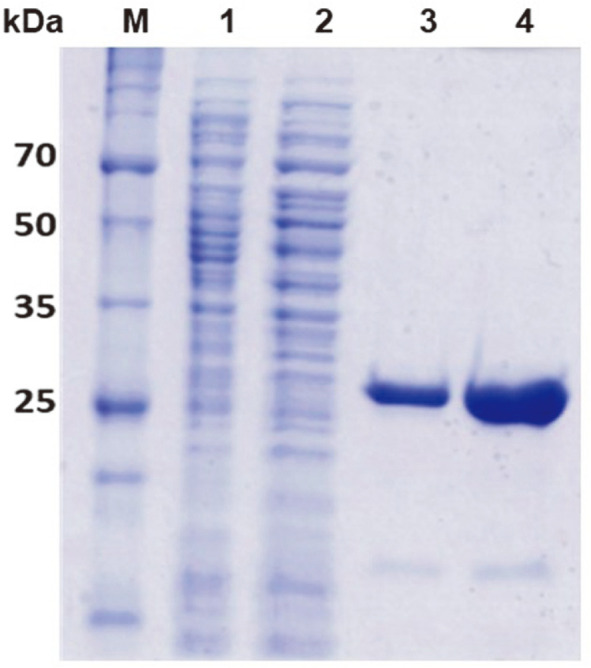
SDS-PAGE analysis of the recombinant Lip29 and Est29 from *G. thermocatenulatus*. Lane M, molecular size marker; lane 1, cell-free extracts from *E. coli* BL21(DE3) harboring recombinant pET21a::Est29 induced with IPTG; lane 2, cell-free extracts after heat treatment; lane 3 and 4, purified Est29 and Lip29 after Ni-NTA chromatography and dialysis.

The full length ORF genes encoding the putative GDSL family lipase were amplified by PCR with specific primers and the PCR products were cloned into pET-21a vector and sequenced. The constructed plasmids were transformed into *E. coli* BL21(DE3) and the recombinant proteins were expressed by IPTG induction. The recombinant Est29 and Lip29 were successfully purified by nickel affinity chromatography, as verified by SDS-PAGE (Fig. 2). Each purified enzyme showed a single band on SDS-PAGE with molecular mass of approximately 29 kDa. The Est29 and Lip29 were purified 14.4-fold with 1.3% yield and 67.3-fold with 5.8% yield, respectively (Table S1). The specific activity of the recombinant enzymes ranged from 0.92 to 2.27 U/mg.

### Substrate Preference of Est29 and Lip29

**Fig. 3:**
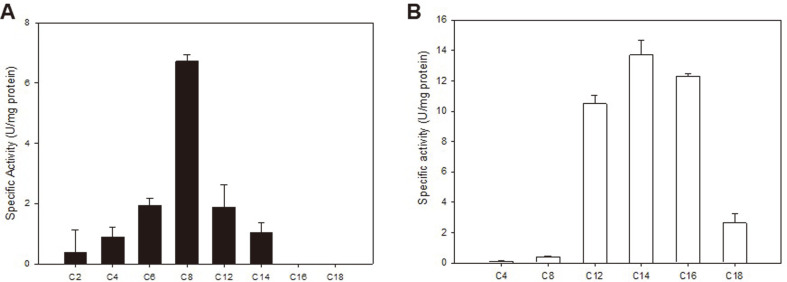
Substrate specificity of recombinant Est29 (A) and Lip29 (B). Reactions were incubated at 55°C (Est29) or 50°C (Lip29) with final substrate concentrations of 10 mM in 50 mM sodium phosphate buffer, pH 6.0 (Est29) or 50 mM bicarbonate-carbonate buffer, pH 9.5 (Lip29). Data are mean values of at least three independent measurements and bars indicate the standard deviation. C2, *p*NP-C2; C4, *p*NP-C4; C6, *p*NP-C6; C8, *p*NP-C8; C12, *p*NP-C12; C14, *p*NP-C14; C16, *p*NP- 16; C18, *p*NP-C18.

Colorimetric protein assay using *p*NP esters consisting different chain lengths was used to examine the substrate preference of both enzymes. The recombinant Est29 exhibited highest activity on short-chain fatty acids in the order of *p*NP-C8, *p*NP-C6 and *p*NP-C4 and no activity was observed on *p*NP-C16 and *p*NP-C18. To date, GDSL family lipolytic enzymes are generally found in extremophiles and these GDSL motif enzymes turned out to be esterases which preferred short-chain fatty acids [8-10]. Therefore, The Est29 is thought to be a typical esterase. On the other hand, the Lip29 exhibited the maximum activity between *p*NP-C12 and *p*NP-C16 and almost no activity toward the short-chain fatty acids below *p*NP-C8, indicating that Lip29 is a lipase, not an esterase (Fig. 3). To our knowledge, there are no reports on detailed characteristics of GDSL-type lipases from thermophilic bacteria.

### Characterization of Est29 and Lip29

**Fig. 4:**
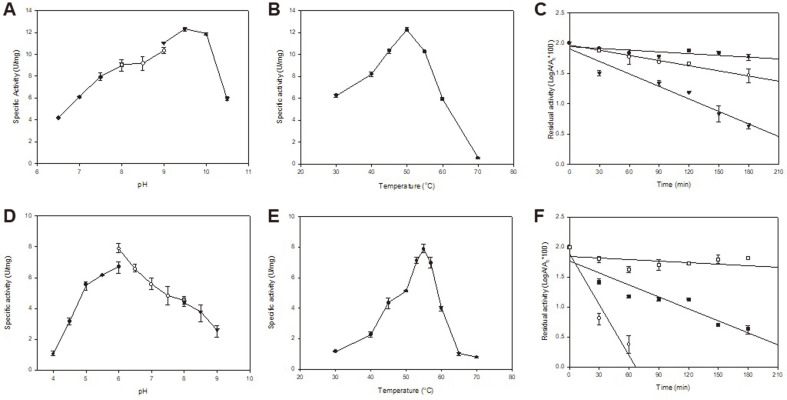
Effects of pH and temperature on the activity of the recombinant Lip29 and Est29. (**A** and **D**) For the determination of optimal pH, activity was measured in sodium acetate buffer (pH 4.0-6.0), sodium phosphate buffer (pH 6.0-8.0), Tris-HCl buffer (pH 8.0-9.0), and bicarbonate-carbonate buffer (pH 9.0- 10.5). (**B** and **E**) For the determination of optimal temperature, activity was measured at pH 9.5 (Lip29) and pH 6.0 (Est29). (**C** and **F**) For the determination of thermostability, the enzyme was incubated in 50 mM bicarbonate-carbonate buffer, pH 9.5 (Lip29) and 50 mM sodium phosphate buffer, pH 6.0 (Est29) at 50°C (closed circles), 60°C (open rectangulars), 65°C (closed rectangulars), 70°C (open circles), 90°C (closed reverse triangles) for 30 min intervals.

The pH and temperature of the recombinant Est29 and Lip29 were determined using *p*NP-C8 and *p*NP-C16 as a substrate, respectively. Lip29 showed an optimum activity at pH 9.5 and maintained more than 70% of activity at pH 9.0 and 10.0 while Est29 was active over a broad range of pH and showed maximum activity at pH 6.0 and above 70% of activity at pH 5.5 and 7.5 (Figs. 4A and 4D). Est29 and Lip29 showed markedly different pH profile against the *p*NP substrate. The purified Est29 and Lip29 exhibited maximum activity at 55°C and 50°C, respectively at their optimum pHs (Figs. 4B and 4E). The activity of both enzymes decreased sharply in temperatures below 40°C and above 60°C. Both enzymes were stable without any significant loss of activity after 3-h incubation at temperature up to 60°C (Figs. 4C and 4F), but the stability of Est29 was sharply dropped at temperature above 65°C and was inactivated completely in about 1 h at 70°C whereas Lip29 was more thermostable than Est29 so that after 3-h incubation at 70°C, around 50% of residual activity was still maintained. The half-life of Lip29 was 108 min at 70°C and 30 min at 90°C, respectively (Fig. 4C).

The effect of metal ions and detergents on enzyme activity was determined using *p*NP-C16 as a substrate (Table 1). The detergents such as DMSO, Triton X-100, Tween 20 did not affect the enzyme activity, but Tween 80 and SDS significantly reduced the activity. Most of the metal ions including Mg^2+^, Co^2+^, Ca^2+^, Mn^2+^, and Fe^3+^ did not affect the enzyme activity at a concentration of 1 mM, however, Cu^2+^ inhibited the activity.

### Modeling Analysis of Lip29

**Fig. 5:**
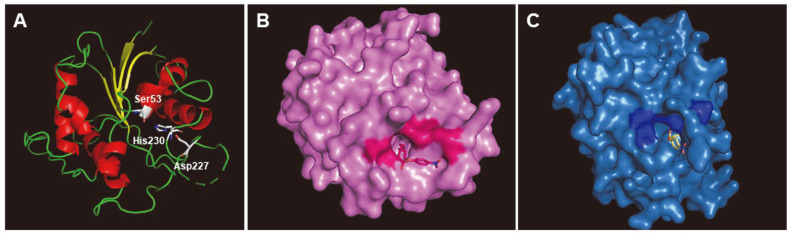
Three-dimensional modeling of Lip29 and Est29. (**A**) Ribbon diagrams of Lip29 shows the overall α/β hydrolase fold. The catalytic triads are shown (Ser53, Asp227, His230). The β-sheets structures and α-helices are colored to yellow and red, respectively. (**B**) Lip29 with the presence of its preferred substrate, *p*NP-C14. The active site is colored to dark pink. (**C**) Est29 with the presence of its preferred substrate, *p*NP-C4. The active site is colored to dark blue.

 To understand how Lip29 exhibit the long-chain substrate preference, the interaction of the model structures of the enzyme with *p*NP substrate was examined using PyRx virtual screening software. PyRx has been used to dock small molecule to a macromolecule like protein to find lead compounds with desired biological function. A computational ligand-target docking approach was used to analyze structural complexes of Lip29 with *p*NP-fatty acid esters to understand the structural basis of this enzyme target specificity. The minimum binding energy indicated that the enzyme was successfully docked with *p*NP substrates. The structural model of Lip29 shows four central β-sheets structures surrounded by seven α-helices (Fig. 5A). The catalytic triad consisting of serine, aspartate and histidine is presented clearly on surface mode structure. The active site is located at the center of the structure, formed a tunnel, channeling to the core (Fig. 5B). Lip29 showed a relatively good binding affinity for long-chain fatty acids as compared to Est29, and conversely, Est29 exhibited a higher binding affinity for short-chain fatty acids. The calculated final docked energies for *p*NP-C14 to Lip29 is -5.9 kcal/mol and for *p*NP-C4 to Est29 is -7.4 kcal/mol. Docking results implies that the active site of Lip29 has the space to allow the long-chain fatty acid substrates, whereas the active site of Est29 is formed a narrow tunnel that hinder long substrates to be catalyzed. The docking results were corresponded well with the three-dimensional modeling of active site in Lip29 and Est29 (Figs. 5B and 5C). The result supports that Lip29 relatively prefers the long-chain substrates as compared to Est29.

## Discussion

*Geobacillus* species including *G. stearothermophilus*, *G. thermoleovorans*, *G. lituanicus*, and *G. thermodenitrificans* have been isolated from several hostile environments including high-temperature oil fields, a corroded pipeline in an extremely deep well, African and Russian hot springs, marine vents, and the Mariana Trench [27, 28]. In the past several years, several thermostable lipases and esterases have been characterized in *Geobacillus* species [14,29-32]. According to their sequences and biochemical properties, these lipolytic enzymes belonged to subfamily I.5 lipases/esterases with conserved GxSxG motif, where first Gly replaced by Ala, and their molecular mass is approximately 43 kDa. A few esterases with GDSL motif from *Geobacillus* species have been characterized [9, 21], but GDSL-type lipases which have preference to long-chain fatty acids have never been identified before. The lipolytic enzymes with GDSL motif have been studied extensively in plants, which are known to be involved in salt stress response and plant immunity reactions [33-35]. Hence, the investigation of microbial diversity has the potential to discover novel lipases and gain better understanding of lipases belonged to GDSL family.

Although the genes presumed to encode a class II lipase in the GDSL family have been identified in the genomes of various *Geobacillus* species, their properties have not yet been characterized. In this study, we expressed two lipolytic enzymes, Est29 and Lip29, from *Geobacillus thermocatenulatus* and characterized their activities against *p*NP ester substrates. Est29 had maximum activity at 55°C and pH 6.0, was stable without any significant loss of activity for 3-h incubation at temperature up to 60°C. The enzyme showed the lipolytic activities broadly from C2 to C14 substrate length with the highest activity on C8. It exhibited very low levels (< 10%) of activity toward the long-chain (C16 and C18) substrates, indicating that it is close to esterase-type enzyme. Similarly, the optimal substrate for EstL5 from *G. thermodenitrificans* T2 was *p*NP-C4 [36]. The thermophilic esterases from *Fervidobacterium nodosum* Rt17-B1 and *Bacillus* sp. K91 also preferred *p*NP-C2 as substrate [37]. However, Lip29 showed strong lipolytic activity to long-chain fatty acids (C12-C16). The substrate specificity of Lip29 was quite different from most GDSL family esterases/lipases from thermophilic origin (Table 2).

Lip29 showed a high stability and optimal activity at high temperatures. Furthermore, the enzyme does not require cofactors and are stable against most detergents and organic solvents. These properties are important feature for industrial applications. Transition metal ions such as Fe^3+^, Co^2+^, and Cu^2+^ have the property of lowering the activity of an enzyme because they bind to the enzyme thereby reduce the stability through conformational change of the enzyme [38]. However, Fe^3+^ and Co^2+^ did not decrease the lipase activity in Lip29. Ca^2+^ has been reported to have a positive effect on activity and stability of family I lipases, however, no significant change was observed in *G. thermocatenulatus* Lip29 [39].

The structure modeling of Lip29 and Est29 was conducted to predict the active site of both enzymes. Lip29 exhibited a typical α/β hydrolase fold which is conserved in SGNH hydrolases and the overall shape of the enzyme was similar with lysophospholipase TesA of *P. aeruginosa* (PDB: 4JGG) which was characterized as a GDSL esterase [13]. Lip29 and Est29 had substrate binding pocket connected behind the active site like TesA. The substrate binding pocket of Est29 was narrower than that of Lip29. The narrow active site may be the reason for the specificity of Est29 on short chain length substrates. This hypothesis was supported by the enzyme-substrate docking analysis. From the docking analysis, the binding energies for *p*NP substrates indicate that the active site of Lip29 prefers long chain length substrates, whereas Est29 prefers short chain length substrates.

In this study, we present the thermostable GDSL-type lipase at which the enzyme was expressed and purified in *E. coli* and characterized. Lip29 is the first GDSL motif enzyme from thermophilic bacteria that has been characterized. It is interesting why the substrate preference between Lip29 and Est29 is different in spite of that the homology of the amino acid sequence of Lip29 with Est29 and other thermostable esterases was very high. In the near future, the availability of the three-dimensional structures of both enzymes would contribute to the understanding of the different substrate specificity.

## Supplemental Materials



Supplementary data for this paper are available on-line only at http://jmb.or.kr.

## Figures and Tables

**Table 1:** Effect of different cations and detergents on Lip29 activity.

Compounds	Specific activity (U/mg)	Relative activity (%)
None	10.7 ± 0.21	100
MgSO_4_	10.4 ± 0.62	98
MgCl_2_	10.1 ± 0.27	95
KCl	10.5 ± 0.29	98
NaCl	10.2 ± 0.19	97
MnCl_2_	9.6 ± 0.45	90
CaCl_2_	10.1 ± 0.12	94
CuCl_2_	1.1 ± 0.15	10
FeCl_3_	9.1 ± 0.05	85
C°Cl_2_	10.4 ± 0.50	97
ZnCl_2_	8.0 ± 0.46	82
SDS	3.0 ± 0.22	24
DMSO	12.2 ± 0.10	99
Triton X-100	11.2 ± 0.98	91
Tween 80	5.9 ± 0.36	48
Tween 20	10.8 ± 0.12	88

Activity is expressed as mean ± standard deviation from three independent experiments.

The cation and detergent concentration used in this study was 1 mM and 1%, respectively.

**Table 2:** Comparison of the recombinant Est29 and Lip29 with other thermostable bacterial GDSL family esterases/lipases.

Enzyme	Type	T_opt_ (°C)	pH_opt_	M.W. (kDa)	Substrate preference	Reference
Rv1075	Esterase	45	9	32.8	C2	[11]
Est8	Esterase	50	9	24.5	C2	[40]
Est19	Esterase	60	9	29.73	C2	[37]
FNE	Esterase	75	8.5	27.5	C2	[41]
Axe2	Esterase	50-60	8.5	24.77	C2	[21]
Xv_EstE	Esterase	44	-	67	C2-C6	[42]
EstL5	Esterase	60	8	-	C4	[9]
Est29	Esterase	55	6	29	C8	This study
Lip29	Lipase	50	9.5	29	C12	This study

## References

[1] Chahinian H, Sarda L (2009). Distinction between esterases and lipases: Comparative biochemical properties of sequence-related carboxylesterases. Protein Pept. Lett..

[2] Arpigny JL, Jaeger KE (1999). Bacterial lipolytic enzymes: classification and properties. Biochem. J..

[3] Bornscheuer UT (2002). Microbial carboxyl esterases: classification, properties and application in biocatalysis. FEMS Microbiol. Rev..

[4] Kovacic F, Babic N, Krauss U, Jaeger K-E, Rojo F (2018). Classification of lipolytic enzymes from bacteria. Aerobic Utilization of Hydrocarbons, Oils and Lipids.

[5] Upton C, Buckley JT (1995). A new family of lipolytic enzymes?. Trends Biochem. Sci..

[6] Akoh CC, Lee GC, Liaw YC, Huang TH, Shaw JF (2004). GDSL family of serine esterases/lipases. Prog. Lipid. Res..

[7] Reina JJ, Guerrero C, Heredia A (2007). Isolation, characterization, and localization of AgaSGNH cDNA: a new SGNH-motif plant hydrolase specific to *Agave americana* L. leaf epidermis. J. Exp. Bot..

[8] Wicka M, Wanarska M, Krajewska E, Pawlak-Szukalska A, Kur J, Cieslinski H (2016). Cloning, expression, and biochemical characterization of a cold-active GDSL-esterase of a *Pseudomonas* sp. S9 isolated from Spitsbergen island soil. Acta Biochim. Pol..

[9] Yang Z, Zhang Y, Shen T, Xie Y, Mao Y, Ji C (2013). Cloning, expression and biochemical characterization of a novel, moderately thermostable GDSL family esterase from *Geobacillus thermodenitrificans* T2. J. Biosci. Bioeng..

[10] Shakiba MH, Ali MS, Rahman RN, Salleh AB, Leow TC (2016). Cloning, expression and characterization of a novel coldadapted GDSL family esterase from *Photobacterium* sp. strain J15. Extremophiles.

[11] Yang DHX, Li S, Liu J, Stabenow J, Zalduondo L, White S, Kong Y (2019). Rv1075c of *Mycobacterium tuberculosis* is a GDSL-like esterase and is important for intracellular survival. J. Infect. Dis..

[12] Kaur J, Kaur J (2019). Rv0518, a nutritive stress inducible GDSL lipase of *Mycobacterium tuberculosis*, enhanced intracellular survival of bacteria by cell wall modulation. Int. J. Biol. Macromol..

[13] Kovacic F, Granzin J, Wilhelm S, Kojic-Prodic B, Batra-Safferling R, Jaeger KE (2013). Structural and functional characterisation of TesA - a novel lysophospholipase A from *Pseudomonas aeruginosa*. PLoS One.

[14] McMullan G, Christie JM, Rahman TJ, Banat IM, Ternan NG, Marchant R (2004). Habitat, applications and genomics of the aerobic, thermophilic genus *Geobacillus*. Biochem. Soc. Trans..

[15] Counago R, Shamoo Y (2005). Gene replacement of adenylate kinase in the gram-positive thermophile *Geobacillus stearothermophilus* disrupts adenine nucleotide homeostasis and reduces cell viability. Extremophiles.

[16] Cripps RE, Eley K, Leak DJ, Rudd B, Taylor M, Todd M (2009). Metabolic engineering of *Geobacillus thermoglucosidasius* for high yield ethanol production. Metab. Eng..

[17] Tabachnikov O, Shoham Y (2013). Functional characterization of the galactan utilization system of *Geobacillus stearothermophilus*. FEBS J..

[18] Rua ML, Atomi H, Schmidt-Dannert C, Schmid RD (1998). High-level expression of the thermoalkalophilic lipase from *Bacillus thermocatenulatus* in *Escherichia coli*. Appl. Microbiol. Biotechnol..

[19] Quyen DT, Schmidt-Dannert C, Schmid RD (2003). High-level expression of a lipase from *Bacillus thermocatenulatus* BTL2 in *Pichia pastoris* and some properties of the recombinant lipase. Protein Exp. Purif..

[20] Ewis HE, Abdelal AT, Lu CD (2004). Molecular cloning and characterization of two thermostable carboxyl esterases from *Geobacillus stearothermophilus*. Gene.

[21] Alalouf O, Balazs Y, Volkinshtein M, Grimpel Y, Shoham G, Shoham Y (2011). A new family of carbohydrate esterases is represented by a GDSL hydrolase/acetylxylan esterase from *Geobacillus stearothermophilus*. J. Biol. Chem..

[22] Bradford MM (1976). A rapid and sensitive method for the quantitation of microgram quantities of protein utilizing the principle of protein-dye binding. Anal. Biochem..

[23] Beisson F, Tiss A, Riviere C, Verger R (2000). Methods for lipase detection and assay: a critical review. Eur. J. Lipid Sci. Tech..

[24] Tang L, Xia Y, Wu X, Chen X, Zhang X, Li H (2017). Screening and characterization of a novel thermostable lipase with detergentadditive potential from the metagenomic library of a mangrove soil. Gene.

[25] Kelley LA, Mezulis S, Yates CM, Wass MN, Sternberg MJE (2015). The Phyre2 web portal for protein modeling, prediction and analysis. Nat. Protoc..

[26] Dallakyan S, Olson AJ (2015). Small-molecule library screening by docking with PyRx. Methods Mol. Biol..

[27] Wissuwa J, Stokke R, Fedøy A-E, Lian K, Smalås AO, Steen IH (2016). Isolation and complete genome sequence of the thermophilic *Geobacillus* sp. 12AMOR1 from an Arctic deep-sea hydrothermal vent site. Stand. Genomic Sci..

[28] Nazina TN, Tourova TP, Poltaraus AB, Novikova EV, Grigoryan AA, Ivanova AE (2001). Taxonomic study of aerobic thermophilic bacilli: descriptions of *Geobacillus subterraneus* gen. nov., sp. nov. and *Geobacillus uzenensis* sp. nov. from petroleum reservoirs and transfer of *Bacillus stearothermophilus*, *Bacillus thermocatenulatus*, *Bacillus thermoleovorans*, *Bacillus kaustophilus*, *Bacillus thermodenitrificans* to *Geobacillus* as the new combinations *G. stearothermophilus*, *G. thermocatenulatus*, *G. thermoleovorans*, *G. kaustophilus*, *G. thermoglucosidasius* and *G. thermodenitrificans*. Int. J. Syst. Evol. Microbiol..

[29] Choi WC, Kim MH, Ro HS, Ryu SR, Oh TK, Lee JK (2005). Zinc in lipase L1 from *Geobacillus stearothermophilus* L1 and structural implications on thermal stability. FEBS Lett..

[30] Leow TC, Rahman RN, Basri M, Salleh AB (2007). A thermoalkaliphilic lipase of *Geobacillus* sp. T1. Extremophiles.

[31] Tayyab M, Rashid N, Akhtar M (2011). Isolation and identification of lipase producing thermophilic *Geobacillus* sp SBS-4S: cloning and characterization of the lipase. J. Biosci.Bioeng..

[32] Zhu YB, Li HB, Ni H, Xiao AF, Li LJ, Cai HN (2015). Molecular cloning and characterization of a thermostable lipase from deep-sea thermophile *Geobacillus* sp. EPT9. World J. Microbiol. Biotechnol..

[33] Ling H, Zhao J, Zuo K, Qiu C, Yao H, Qin J (2006). Isolation and expression analysis of a GDSL-like lipase gene from *Brassica napus* L. J. Biochem. Mol. Biol..

[34] Naranjo MA, Forment J, Roldan M, Serrano R, Vicente O (2006). Overexpression of *Arabidopsis thaliana* LTL1, a salt-induced gene encoding a GDSL-motif lipase, increases salt tolerance in yeast and transgenic plants. Plant Cell Environ..

[35] Hong JK, Choi HW, Hwang IS, Kim DS, Kim NH, Choi DS (2008). Function of a novel GDSL-type pepper lipase gene, CaGLIP1, in disease susceptibility and abiotic stress tolerance. Planta.

[36] Yang Z, Zhang Y, Qiu R, Huang J, Ji C (2013). Crystallization and preliminary X-ray diffraction analysis of a thermostable GDSLfamily esterase, EstL5, from *Geobacillus thermodenitrificans* T2. Acta Crystallogr. Sect. F Struct. Biol. Cryst. Commun..

[37] Yu T, Ding J, Zheng Q, Han N, Yu J, Yang Y (2016). Identification and characterization of a new alkaline SGNH hydrolase from a thermophilic bacterium *Bacillus* sp. K91. J. Microbiol. Biotechnol..

[38] Rahman RN, Baharum SN, Basri M, Salleh AB (2005). High-yield purification of an organic solvent-tolerant lipase from *Pseudomonas* sp. strain S5. Anal. Biochem..

[39] Kambourova M, Kirilova N, Mandeva R, Derekova A (2003). Purification and properties of thermostable lipase from a thermophilic *Bacillus stearothermophilus* MC 7. J. Mol. Catal. B Enzym..

[40] Ding J, Yu T, Liang L, Xie Z, Yang Y, Zhou J (2014). Biochemical characterization of a GDSL-motif esterase from *Bacillus* sp. K91 with a new putative catalytic mechanism. J. Microbiol. Biotechnol..

[41] Yu S, Zheng B, Zhao X, Feng Y (2010). Gene cloning and characterization of a novel thermophilic esterase from *Fervidobacterium nodosum* Rt17-B1. Acta Biochim. Biophys. Sin. (Shanghai).

[42] Talker-Huiber D, Jose J, Glieder A, Pressnig M, Stubenrauch G, Schwab H (2003). Esterase EstE from *Xanthomonas vesicatoria* (Xv_EstE) is an outer membrane protein capable of hydrolyzing long-chain polar esters. Appl. Microbiol. Biotechnol..

