# Supplementary material for: Identification and Characterization of a Novel Thermostable GDSL-Type Lipase from Geobacillus thermocatenulatus
Source: J Microbiol Biotechnol. 2021 Feb 12;31(3):483–91. doi: 10.4014/jmb.2012.12036 (PMC9706006; doi:10.4014/jmb.2012.12036)
Supplement: Supplementary file 1 [file jmb-31-3-483-supple.pdf]

Supplementary Table S1. Purification step of the recombinant Est29 and Lip29

	Total activity (U)[a]	Total protein (mg)[b]	Specific activity (U/mg)	Yield (%)	Purification (-fold)
Est29					
Cell-free extract	96.3	1505.1	0.06	100	1.0
Heat treatment	78.3	388.2	0.20	81.4	3.2
Ni-NTA	1.2	1.3	0.92	1.3	14.4
Lip29					
Cell-free extract	36.7	1087.0	0.03	100	1.0
Heat treatment	11.9	293.1	0.04	32.5	1.2
Ni-NTA	2.1	0.9	2.27	5.8	67.3

[a]The activity unit is expressed as μmol/min with *p*NP-C16 as substrate.

[b]The amount of total protein was determined by the Bradford assay using BSA as a standard.
